# Oral Microbiome and Gingival Gene Expression of Inflammatory Biomolecules With Aging and Periodontitis

**DOI:** 10.3389/froh.2021.725115

**Published:** 2021-09-17

**Authors:** Jeffrey L. Ebersole, Radhakrishnan Nagarajan, Sreenatha Kirakodu, Octavio A. Gonzalez

**Affiliations:** ^1^Department of Biomedical Sciences, School of Dental Medicine, University of Nevada Las Vegas, Las Vegas, NV, United States; ^2^Center for Oral Health Research, College of Dentistry, University of Kentucky, Lexington, KY, United States; ^3^Center for Oral and Systemic Health, Marshfield Clinic Research Institute, Marshfield Clinic Health System, Marshfield, WI, United States; ^4^Division of Periodontology, College of Dentistry, University of Kentucky, Lexington, KY, United States

**Keywords:** non-human primate, aging, microbiome, periodontitis, transcriptome

## Abstract

Although data describe the presence and increase of inflammatory mediators in the local environment in periodontitis vs. health in humans, details regarding how these responses evolve in the transition from health to disease, changes during disease progression, and features of a resolved lesion remain unknown. This study used a nonhuman primate model of ligature-induced periodontitis in young, adolescent, adult, and aged animals to document features of inflammatory response affected by age. Rhesus monkeys had ligatures tied and provided gingival tissue biopsy specimens at baseline, 0.5, 1, and 3 months of disease and at 5 months of the study, which was 2 months post-ligature removal for clinically resolved tissues. The transcriptome was assessed using microarrays for chemokine (*n* = 41), cytokine (*n* = 45), chemokine receptor (*n* = 21), cytokine receptor (*n* = 37), and lipid mediator (*n* = 31) genes. Limited differences were noted in healthy tissues for chemokine expression with age; however, chemokine receptor genes were decreased in young but elevated in aged samples. IL1A, IL36A, and IL36G cytokines were decreased in the younger groups, with IL36A elevated in aged animals. IL10RA/IL10RB cytokine receptors were altered with age. Striking variation in the lipid mediator genes in health was observed with nearly 60% of these genes altered with age. A specific repertoire of chemokine and chemokine receptor genes was affected by the disease process, predominated by changes during disease initiation. Cytokine/cytokine receptor genes were also elevated with disease initiation, albeit IL36B, IL36G, and IL36RN were all significantly decreased throughout disease and resolution. Significant changes were observed in similar lipid mediator genes with disease and resolution across the age groups. Examination of the microbiome links to the inflammatory genes demonstrated that specific microbes, including *Fusobacterium, P. gingivalis, F. alocis, Pasteurellaceae*, and *Prevotella* are most frequently significantly correlated. These correlations were generally positive in older animals and negative in younger specimens. Gene expression and microbiome patterns from baseline were distinctly different from disease and resolution. These results demonstrate patterns of inflammatory gene expression throughout the phases of the induction of a periodontal disease lesion. The patterns show a very different relationship to specific members of the oral microbiome in younger compared with older animals.

## Introduction

Periodontal lesions and the associated hard and soft tissue destruction of the periodontium represent the outcomes of a chronic inflammatory response to the burden of the microbiome at affected sites. This disease is accentuated with age, likely reflecting a combination of general tissue health and remodeling capabilities, long-term environmental and epigenetic effects on tissue homeostasis and the oral microbiome, and immunosenescent changes in the host response profile [1]. This dysbiotic microbiome displays an altered presence and an abundance of microbial members, selected microorganisms that appear to directly facilitate changes in the local environment enhancing a more pathogenic microbiome, and altered gene expression profiles of normal commensal bacteria that can contribute to altering the host-inflammatory response [2–5]. Approaches to lessen this microbial burden through both mechanical and chemical strategies generally show a high frequency of positive responses across the population in decreasing the continuation of these lesions [6–8]. Substantial literature regarding details of the human microbiome and different arrays of host responses in the juxtaposed tissues and gingival crevicular fluid exists, attempting to establish relationships between the microbiome component bacteria and/or complexes of these bacteria that dysregulate the host responses and likely drive the destructive inflammation [9–13].

Nevertheless, a challenge with the investigations of naturally occurring periodontitis in humans is that the biology of the disease sites is only studied at the time of clinically detected lesions, using somewhat crude observational tools for inflammation and tissue destruction. As such, we have employed a non-human primate model of naturally occurring and experimental ligature-induced periodontitis for decades. The disease has been shown to affect a wide array of non-human primate species that demonstrate clinical, microbiological, and immunological features similar to human disease [14–16]; exhibit both age and sex differences similar to humans [17, 18]; can be altered by mechanical, chemical, and vaccination interventions [19–23]; and enable detailed studies of clinical resolution of periodontal lesions. We have recently reported on gene expression profiles in gingival tissues that enable discrimination of health, disease kinetics, and resolution of the lesions [24]. Within this array of differentially expressed genes, we identified members of the chemokine and cytokine families, and cell receptors that contributed to the phase assignment.

This analysis examined gingival tissue responses and microbiome features in non-human primates of different ages. The interrogation of the dataset focused on a wide array of chemokines, cytokines, chemokine and cytokine receptors, and genes related to inflammatory lipid mediator production and control. Additionally, the data enable us to explore the relationship of specific microbes and complexes that appear to be more directly linked to the altered expression of these host-response biomarkers in health and disease across the lifespan.

## Materials and Methods

### Animals and Diet

Rhesus monkeys (*Macaca mulatta*) (*n* = 36; 17 male, 19 female) housed at the Caribbean Primate Research Center at Sabana Seca, Puerto Rico, were examined for periodontal health [18, 25, 26] and were used to determine the results from a ligature-induced periodontitis model. Young (≤3 years), adolescent (3–7 years), adult (12–16 years), and aged (18–23 years) were used in the study with nine animals/group. As an estimate, 1 monkey year approximates 3.5 human years; thus, the groups represented human subjects about 6–9 yo, 11–25 yo, 42–56 yo, 63–82 yo. The non-human primates were fed a 20% protein, 5% fat, and 10% fiber commercial monkey diet (diet 8773, Teklad NIB primate diet modified: Harlan Teklad, Madison, WI). The diet was supplemented with fruits and vegetables, and water was provided *ad libitum* in an enclosed corral setting.

As we have reported previously, the protocol was approved by the Institutional Animal Care and Use Committees (IACUC) of the University of Puerto Rico and the University of Kentucky, and a ligature disease model was utilized [27]. The clinical examination included probing pocket depth (PPD) and bleeding on probing (BOP; 0–5 scale) [28]. Periodontal health was defined by mean PPD ≤ 3.0 mm and mean BOP ≤ 1 (0–5 scale) in a full mouth examination excluding 3rd molars and canines [27]. Ligature-induced periodontal disease was initiated, as we have previously reported [27], and gingival and subgingival plaque samples were taken at 0.5, 1, and 3 months (initiation/progression), and 2 months after the removal of ligatures and local factors (resolution). Determination of periodontal disease at the sampled site was documented by assessment of the presence of BOP and PPD of >4 mm, as we have described previously [26]. Changes in these clinical measures of BOP and PPD in health, during disease initiation and progression, and resolution in these age groups have been described previously [24]. Briefly, all animals demonstrated significant increases in BOP within 0.5 months (0.6 ± 0.1 to 3.8 ± 0.1; mean ± SEM), with somewhat elevated levels in the younger age groups. PPD increases were noted in all animals across all age groups with peak disease at 1–3 months. However, in both young and adolescent animals, the PPD measures for the rate of change and peak levels of the disease were less than in the adult and the aged group (young/adolescent: 1.4 ± 0.1 to 3.9 ± 0.2; adult/aged: 2.7 ± 0.1 to 5.3 ± 0.2). The clinical resolution of the sites occurred over 2 months, resulting only from the removal of the ligatures. At resolution, both BOP and PPD measures decreased across all age groups, albeit generally remaining above measures for the baseline, healthy tissues.

### Microbiome Analysis

Subgingival bacterial samples were obtained from the 36 animals by a curette and analyzed using a MiSeq instrument [29, 30] for the total composition of the microbiome from each sample [31, 32]. Sequences were clustered into phylotypes based on their sequence similarity, and these binned phylotypes were assigned to their respective taxonomic classification using the Human Oral Microbiome Database (HOMD V13) (http://www.homd.org/index.php?name=seqDownload&file&type=R), as we have described previously [29]. Raw data were deposited at the NIH NCBI (BioProject ID PRJNA516659). Statistical differences of bacterial OTUs were determined with a *t-test* (*p* < 0.05). Correlations of OTUs within the oral microbiome were determined using a Pearson correlation coefficient analysis (*p* < 0.05). Correlations between the microbiome components and the gingival gene expression were determined only for matching samples derived from the same tooth in each of the animals. Matching samples with sufficient microbiome signals were compared for 46 samples in adults and 25 samples from the young group obtained at health and throughout the ligature model. As we have reported previously [29], of up to 394 OTUs identified in the non-human primate oral samples, the targeted OTU selection for this study was 58 for the adult/aged samples in which the relative abundance of these OTUs encompassed 74–82% of the signal and 49 OTUs in the young/adolescent samples covering 77–86% across the samples.

### Gingival Tissue Sample Collection and mRNA Analysis

Ligated teeth included the 1st premolar and 1st and 2nd molars in each of the four quadrants. Gingival tissue samples from healthy, diseased, and resolved sites were surgically collected using a standard gingivectomy technique (e.g., crevicular incision followed by an interdental incision at the base of the papillae), providing a gingival sample from each animal at each time point, and total RNA was extracted for microarray analysis [26]. Each animal provided a gingival tissue sample at baseline, during the three disease times, and at resolution. All of these were obtained from distinct unique sites at each time point. The analytic hybridization was to the GeneChip® Rhesus Gene 1.0 ST Array (Affymetrix, Santa Clara, CA, USA) for the ligature-induced periodontitis model, similar to methods we have described previously [25, 26, 33–35].

### Data Analysis

Chemokine (*n* = 41), cytokine (*n* = 45), chemokine receptors (*n* = 21), cytokine receptors (*n* = 37), and lipid mediator (*n* = 31) genes were targeted in the analysis (Supplementary Table 1). Beyond the specific Affymetrix probe annotation provided by the company, we annotated within the GeneChip® Rhesus Gene 1.0 ST Array additional probes for the host genes in this microarray. These included unannotated probes, whereby the nucleotide base sequence (https://www.affymetrix.com/analysis/index.affx#1_2) for each probe ID was subjected to a Blast (https://blast.ncbi.nlm.nih.gov/Blast.cgi) query that identified the *M. mulatta* gene ID with the greatest percent identified for the specific sequence. We selected the most targeted gene ID that always showed >90% identity for annotating the gene list for the analysis.

The expression intensities across the samples were estimated using the robust multiarray average (RMA) algorithm with probe-level quintile normalization, as implemented in the Partek Genomics Suite software version 6.6 (Partek, St. Louis, MO). The differential expression was initially compared using one-way ANOVA across time points within an age group. For genes that had significant mean differences, two-sample *t*-tests were used to investigate differences comparing baseline healthy to disease and resolution samples. Statistical significance was considered by a *p*-value < 0.05 adjusted for the number of correlations tested using a Benjamini-Hochberg procedure. The data have been uploaded into GEO accession GSE180588 (https://www.ncbi.nlm.nih.gov/gds).

Correlation analyses were determined using a Pearson correlation coefficient with a *p*-value < 0.05 and adjusted for the number of correlations tested. Gephi software was used for generating a dual-circle layout representing significant correlations (α = 0.01) between inflammatory mediator genes and the microbiome OTUs. A parametric *t*-test (α = 0.05) was used to identify the differentially expressed genes and bacteria at 0.5, 1, 3, and 5 months using baseline expression as the control. Those that were differentially expressed, at least at one of the time points, were identified and a principal component analysis (PCA) was subsequently used to examine clustering of both age groups of the animals across the different time points from their normalized expression profiles.

## Results

### Age Effects on Inflammatory Gene Expression in Healthy Gingival Tissues

**Figure 1:**
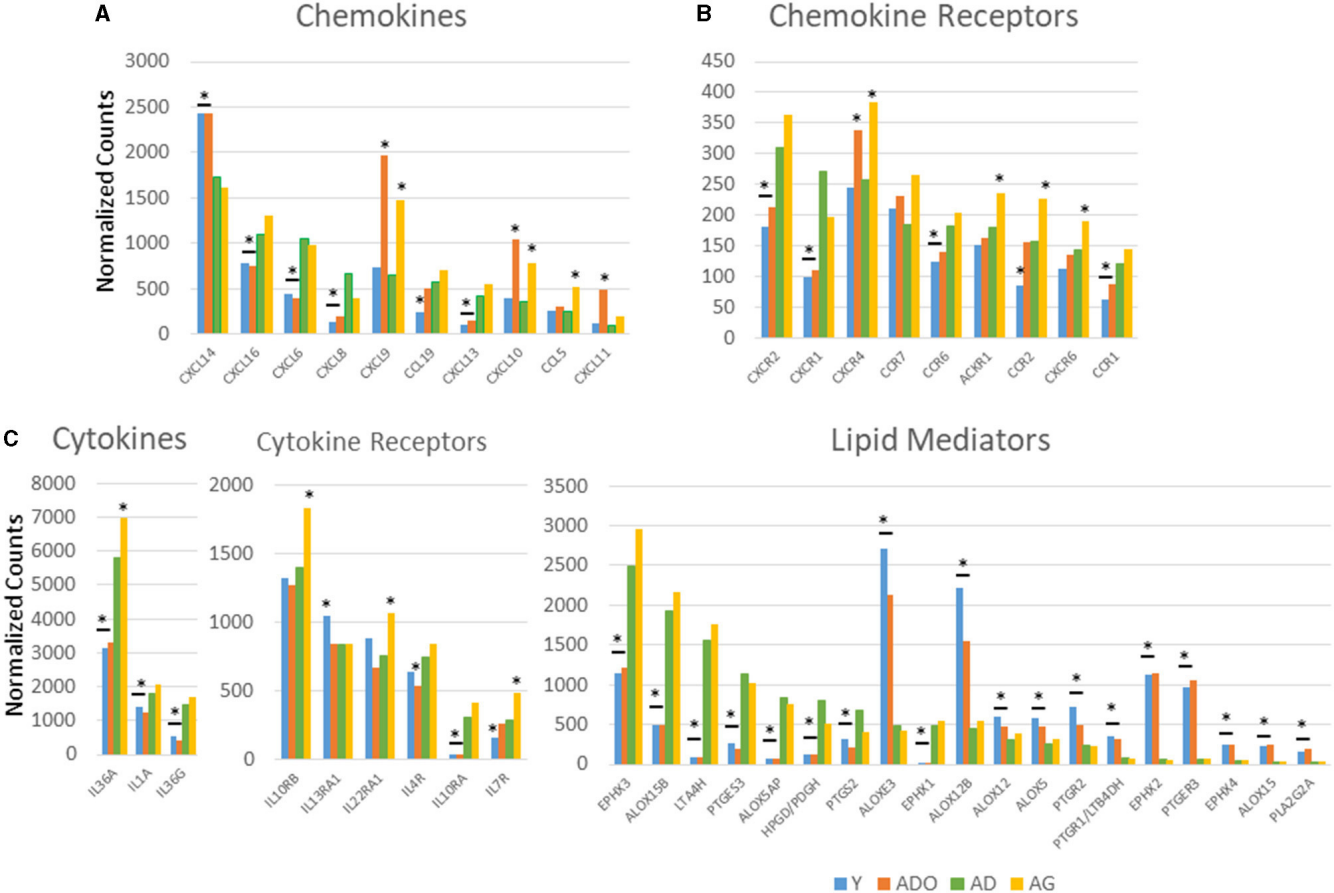
Comparison of normalized expression values for chemokine and chemokine receptor **(A)**, cytokine and cytokine receptor **(B)**, and lipid mediator **(C)** genes in healthy gingival tissues across the age groups. Depicted are those genes in each category with a significant difference among the groups based on adjusted ANOVA analysis (*p* < 0.05). The bars represent mean normalized expression values for each age group of nine animals. The bar and/or asterisk denote significant difference from the adult group levels.

Figure 1 displays a comparison of the normalized counts for gene expression in baseline healthy tissue samples from the different age groups for those genes with significant differences across the age groups. A rather limited repertoire of chemokine genes varied across the age groups, with CXCL16, CXCL6, CXCL8, CCL19, and CXCL13 being decreased in the young and/or adolescent samples. In contrast, CXCL14, CXCL9, and CXCL10 were increased in the adolescent and aged samples, and CXCL11 only in the adolescent samples. Differential expression was more pronounced with the chemokine receptor levels with CXCR2/IL8RB, CXCR1, CCR6, and CCR1 all decreased in the young and adolescent animals, and in addition CCR2 in only the young samples. Generally, differential expression in the aged samples was increased including CXCR2, CXCR4, CCR7, ACKR1, and CXCR6. A very limited number of cytokines differed in healthy samples across the age groups with lower levels of IL36A, IL36G, and IL1A in young and adolescent animals and elevated IL36A in the aged healthy tissues. The figure also summarizes cytokine receptor expression. With this set of inflammatory genes, specimens from young and adolescent animals were quite similar to adult levels, except IL10RA that was substantially decreased and IL13RA1 that was increased in the young animals. Elevated levels of expression of IL10RB, IL22RA1, and IL7R were the limited group of receptor genes that were increased in the aged samples. The figure also displays variations in genes related to the production and functional actions of inflammatory lipid mediators. There were striking differences in the expression of an array of these genes compared with the adult levels that focused on young and adolescent gingival tissues. Nine of these genes were decreased compared with adults and nine additional ones were increased. Of particular note were the significant increases in ALOXE3, ALOX12B, EPHX2, and PTGER3 in the younger groups. Thus, an overview of the differences in healthy tissues showed limited effects on the genes for chemokine and cytokine effector molecules affected by aging; however, more numerous effects were noted for receptors for the chemokines and cytokines. In contrast, the gene family associated with inflammatory lipids was substantively different in the young and adolescent groups, suggesting the potential for a very different milieu of these mediators even in healthy tissues. Supplementary Figures 1A–C provides the overall expression profile for all the inflammatory genes organized based upon the expression level in the adult samples.

### Age Effects on Inflammatory Gene Expression and Progressing Periodontitis

**Figure 2:**
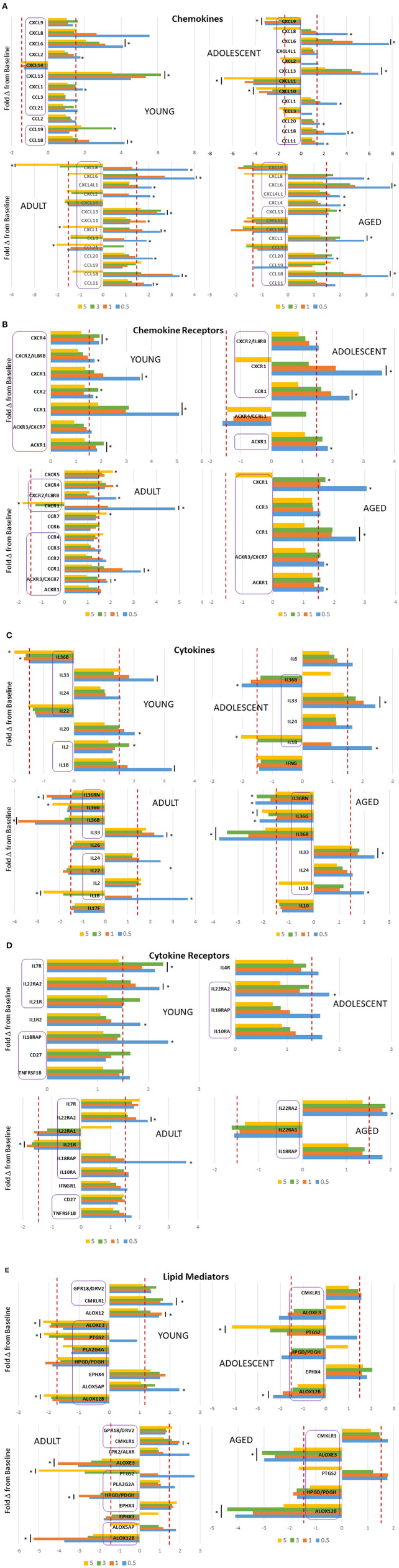
Fold-changes in chemokine **(A)**, chemokine receptor **(B)**, cytokine **(C)**, cytokine receptor **(D)**, and lipid mediator **(E)** genes comparing disease time point, i.e., 0.5, 1, 3 months, and resolution, i.e., 5-month, samples to baseline levels in each age group (nine animals/group). Genes in each category are presented with differential expression of ≥1.5 above or below the baseline. The bars denote mean differential expression at each timepoint and the bars and/or asterisks denote significant difference at *p* < 0.05 (*t* test). The purple boxes highlight the genes that overlap in differential expression across the age groups.

Of particular interest were changes that could occur in the array of inflammatory mediator genes during initiation, progression, and resolution of experimental periodontitis using non-human primates across the lifespan. Figure 2A demonstrates chemokine gene changes with disease affected by age. In the young and adolescent animals, similar chemokines were affected by CXCL1, CXCL2, CXCL6, CXCL8, CXCL13, CXCL14, CCL3, and CCL18 all increased with disease initiation and progression. In contrast, CXCL9, CXCL10, and CXCL11 were all decreased in the adolescent samples. Few of these remained elevated in the resolution samples. Generally, this profile of genes was also differentially expressed in samples from adults and aged animals. Additionally, CCL11, CCL20, and CXCL4L1 were elevated in the adults and aged samples. Also, with these inflammatory molecules, the greatest increase was observed in the disease initiation samples, with CXCL8 decreasing at resolution in all but the young animals. Figure 2B shows the changes occurring in chemokine receptors with the disease. In this case, there was a commonality across all age groups with CCR1, CXCR1, and ACKR1 being increased. As with the chemokines these changes were most greatly affected with disease initiation. Cytokine response profiles (Figure 2C) showed elevations in IL1B, IL36B, IL24, and IL33 in all age groups. While IL1B was primarily increased with disease initiation, IL33 was elevated throughout the disease and in resolution samples, and IL24 only at disease initiation. In contrast, IL36 cytokines were all decreased with disease, and also IL22 in young and adult animals. IL18RAP and IL22RA2 cytokine receptors were elevated across all age groups with the former primarily at disease initiation and the latter throughout the disease and even resolution samples (Figure 2D). Additionally, IL7R was increased in young and adult animals at all disease time points. Within the lipid mediator gene portfolio, CMKLR1 was increased in all age groups, with EPHX4 elevated except in aged samples. ALOX12B, ALOXE3, and HPDG/PDGH were decreased throughout disease and resolution in all age groups. In contrast, PTGS2 was elevated in only the adult and aged animals and was decreased in the younger groups (Figure 2E).

### Oral Microbiome and Inflammatory Gene Expression in Health and Disease

**Figure 3:**
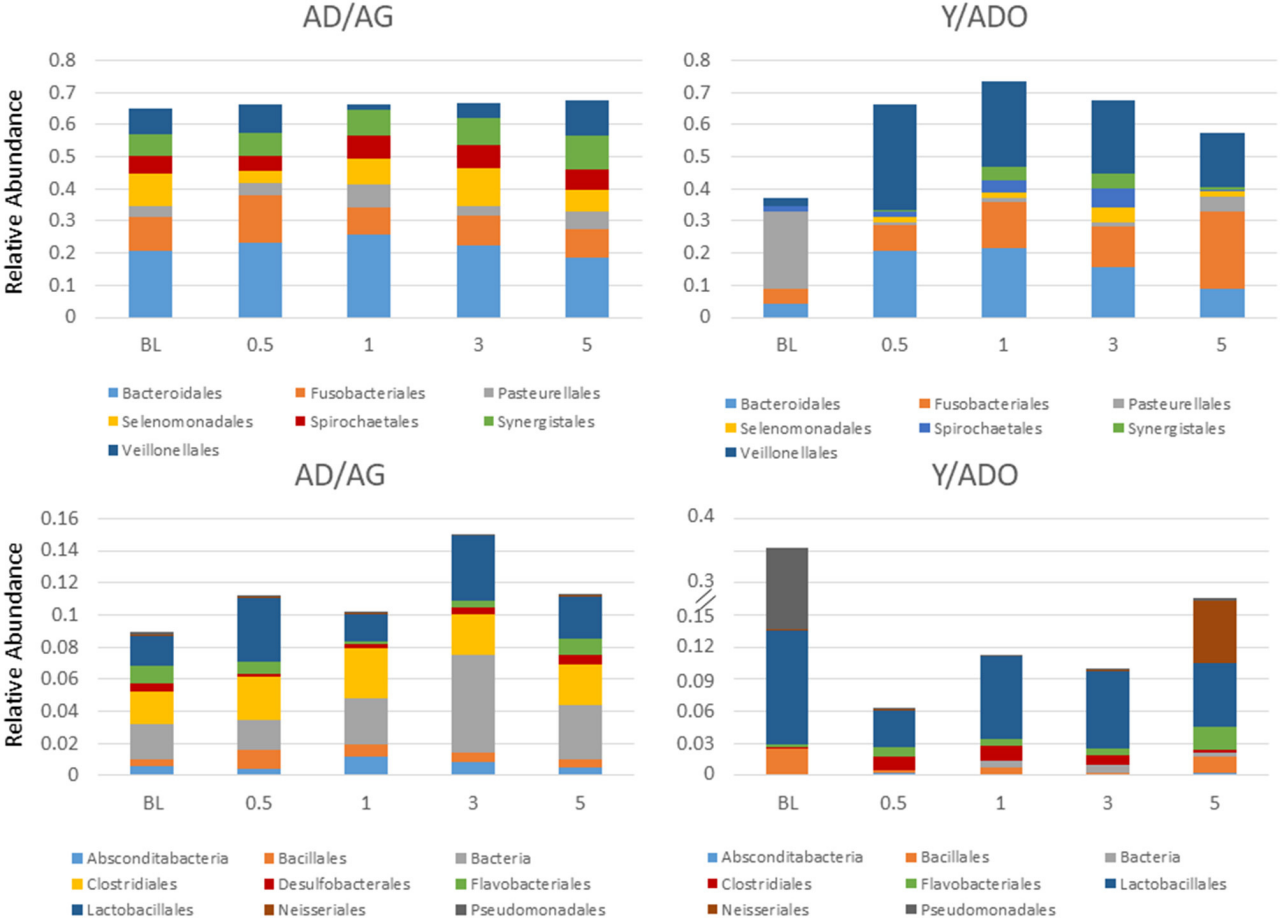
Stacked bar charts display a relative abundance of microbial families within the oral microbiome samples from the young/adolescent (Y/ADO; *n* = 25 samples) and adult/aged (AD/AG; *n* = 46) groups. Abundance levels are grouped in high abundance families (TOP) and low abundance families (BOTTOM) with in the AD/AG samples.

To have sufficient analytic power to estimate correlations of the various inflammatory genes with the overall microbiome characteristics from matched sites, sample results from the adult and aged groups were combined (AD/AG), as were the sample results from the young and adolescent animals (Y/ADO). Figure 3 summarizes the variations in the oral microbiomes of the different age groups grouped into the primary bacterial families that generally comprised 77–86% of the reads in the Y/ADO samples and 74–82% in the AD/AG groups. The results demonstrated somewhat limited differences in the major families in the AD/AG group from health to disease and resolution; however, alterations in the relative abundance of unclassified bacteria, Bacillales, and Lactobacillales were observed with the disease in this group. This contrasted with clear differences in the major families in samples from Y/ADO animals. The data showed a decrease in Pasteurellales and increases in Bacteroidales, Fusobacteriales, Synergistales, and Veillonellales with the disease. Also noted was the substantial increases in the relative abundance of Bacillales, Lactobacillales, and Pseudomonadales in the Y/ADO baseline (healthy) samples compared with the older group. Decreased Lactobacillales and increases in Clostridiales and Flavobacteriales were observed in disease in the younger groups.

Initial correlations of inflammatory mediators and the microbiome were explored with measures of microbiome diversity and richness by determining the Shannon diversity index [36], inverse Simpson value (https://mothur.org/wiki/invsimpson/), Chao1 [37], ACE [38], and each of the inflammatory mediator genes. Table 1 depicts the frequency of these correlations with the various groups of inflammatory genes. The relationships with the chemokine genes were primarily observed with the diversity indices focused on richness and evenness of species (Shannon, inverse Simpson), were skewed toward negative correlations, and primarily occurred in healthy samples from both the age groups. A similar skewing toward negative correlations in both age groups was seen with the cytokine and cytokine receptor genes, although these relationships were only noted in the Y/ADO group healthy samples for the chemokine receptor and lipid mediator genes. Generally, the values for the disease samples across all the gene families were less remarkable with any of these overall microbiome measures. Finally, the ACE measure is an abundant description of coverage that estimates species richness. This measure showed extensive positive correlations with healthy samples from the Y/ADO group. Thus, there appeared to be a more robust relationship between the overall microbiome and these inflammatory mediator transcript levels under conditions of clinical periodontal health; however, with the disease, these relationships appear to disappear, and the host–microbe interactions become more related to individual or complexes of the microbes within the overall microbiome.

**Table 1:** Distribution of significant correlations of genes for various inflammatory mediator families and the individual measures of the overall microbiome features in the different age groups of animals. The values denote the number of positive (Pos) or negative (Neg) correlations.

**Genes**	**Diversity Index**	**AD/AG Health**		**AD/AG Disease**		**Y/ADO Health**		**Y/ADO Disease**	
		**Pos**	**Neg**	**Pos**	**Neg**	**Pos**	**Neg**	**Pos**	**Neg**
Chemokines	Inverse Simpson	1	6	1	2	1	9	0	0
	Shannon	4	4	1	3	1	8	0	0
	Chao1	0	3	0	2	0	7	0	1
	Ace	0	3	0	1	9	2	1	1
Chemokine Receptors	Inverse Simpson	1	3	0	1	0	7	0	1
	Shannon	0	0	0	1	1	8	0	1
	Chao1	0	4	0	1	1	1	0	1
	Ace	0	4	0	1	7	1	0	3
Cytokines	Inverse Simpson	4	4	0	3	2	6	0	0
	Shannon	4	4	0	5	2	8	0	2
	Chao1	2	0	0	3	2	1	0	0
	Ace	0	1	0	3	11	5	1	1
Cytokine Receptors	Inverse Simpson	2	5	0	0	0	6	0	0
	Shannon	0	4	2	0	0	7	1	0
	Chao1	4	2	1	0	2	3	1	0
	Ace	4	1	1	0	8	0	0	2
Lipid Mediators	Inverse Simpson	1	0	0	0	1	2	0	0
	Shannon	1	0	1	1	0	5	0	0
	Chao1	2	1	0	0	3	1	0	0
	Ace	1	0	0	0	5	2	0	0

### Oral Microbiome Components and Inflammatory Gene Expression in Health and Disease

**Figure 4:**
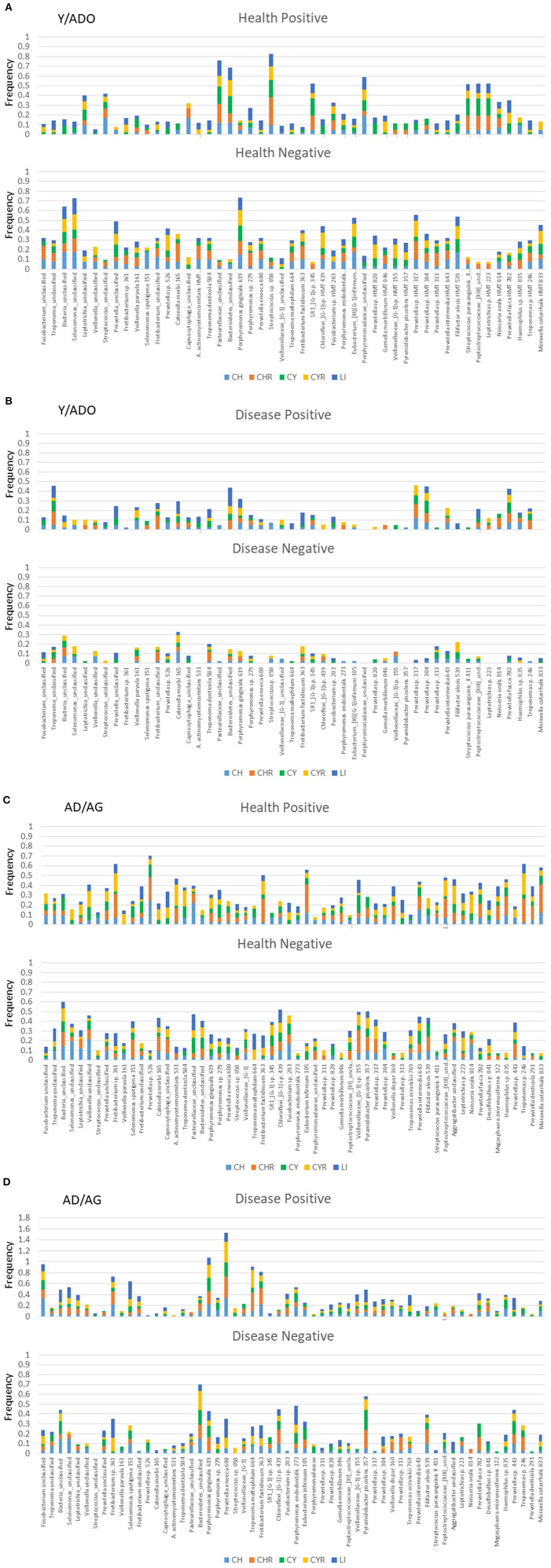
Stacked bars denoted the frequency of genes for the five families of inflammatory mediator genes (CH, chemokines; CHR, chemokine receptors; CY, cytokines; CYR, cytokine receptors; LI, lipid mediators) that correlated significantly with a relative abundance of the individual microbiome components. Summary of results for both positive and negative correlations in healthy **(A)** or diseased **(B)** gingival samples from a pooled group of younger animals and in healthy **(C)** or diseased **(D)** gingival samples from a pooled group of older animals.

Figures 4A,B summarizes the frequency of significant correlations between individual microbiome operational taxonomic units (OTUs) and host chemokine, chemokine receptor, cytokine, cytokine receptor, and lipid mediator genes. Figure 4A depicts the distribution of significant positive or negative gene correlations in healthy samples from the Y/ADO animals. Of interest was a select group of bacteria including, *Pasteurallaceae*_unclassified, *Bacteroidetes*_unclassified, *Streptococcus* sp. HMT 058, and *Porphyromonadaceae*_unclassified, all showing a high frequency of positive correlations with a range of the inflammatory mediator genes. In contrast, Bacteria_unclassified, *Selenomonas*_unclassified, and *P. gingivalis* HMT 619 demonstrated a similar broad relationship with the inflammatory mediator genes, but with a negative correlation. Figure 4B demonstrates a very different profile of correlations in disease samples from the Y/ADO animals. Negative correlations of individual OTUs with any of the groups of inflammatory genes were quite limited in this age group. While not at the same frequency levels as in health, *Treponema*_unclassified, *Bacteroidetes*_unclassified, and *Prevotella sp*. HMT 304 and 313 showed positive correlations with the inflammatory mediators, with some difference in the subset of mediators, mostly related to these OTU levels.

Similar correlations with healthy samples from the AD/AG group are presented in Figure 4C. A large number of the OTUs demonstrated positive or negative correlations with an array of inflammatory mediator genes. In particular, *Fretibacterium* sp. HMT 361, *Prevotella* sp. HMT 526, *F. fastidiosum* HMT 363, *Eubacerium infirmum* 105, and *Treponema* sp. HMT 246 showed a high prevalence of significant positive correlations with some differences in the specific categories of inflammatory mediators represented by these correlations. As was noted with the Y/ADO samples, a different set of OTUs was negatively correlated with panels of the inflammatory mediator genes and included Bacteria_unclassified, *Pasteurellaceae*_unclassified, *Chloroflexi*_{G-1] sp. HMT 439, *Veillonellaceae*_[G-1] sp. HMT 155, and *P. piscolens* HMT 357. The prevalence of positive or negative correlations in the AD/AG samples during disease was more limited across the OTUs, with some showing a clear predisposition for these relationships (Figure 4D). An elevated frequency of significant positive correlations was observed with *Fusobacterium*_unclassified, *Fretibacterium* sp. HMT 361, *S. sputigena* HMT 151, *P. gingivalis* HMT 619, *P. enoeca* HMT 600, *T. maltophilum* HMT 664, and *F. fastidiosum* HMT 363. This contrasted with the negatively correlated OTUs that were dominated by *Bacteroidetes*_unclassified, *P. endodontalis* HMT 273, and *P. piscolens* HTM 357. The principal features of these relationships in both age groups were clear differences in the individual bacteria related to inflammatory mediator gene expression, and also some overlap in the OTUs detected in the young and older age group. Another observation was that although some of these bacteria appeared to show a broad relationship to the array of inflammatory mediator genes, individual bacteria clearly had a predilection for specific groups of chemokines/cytokines, their receptors, or lipid mediators that was most prominent in the older age group.

### Microbial Complexes and Inflammatory Gene Expression in Aging

**Figure 5:**
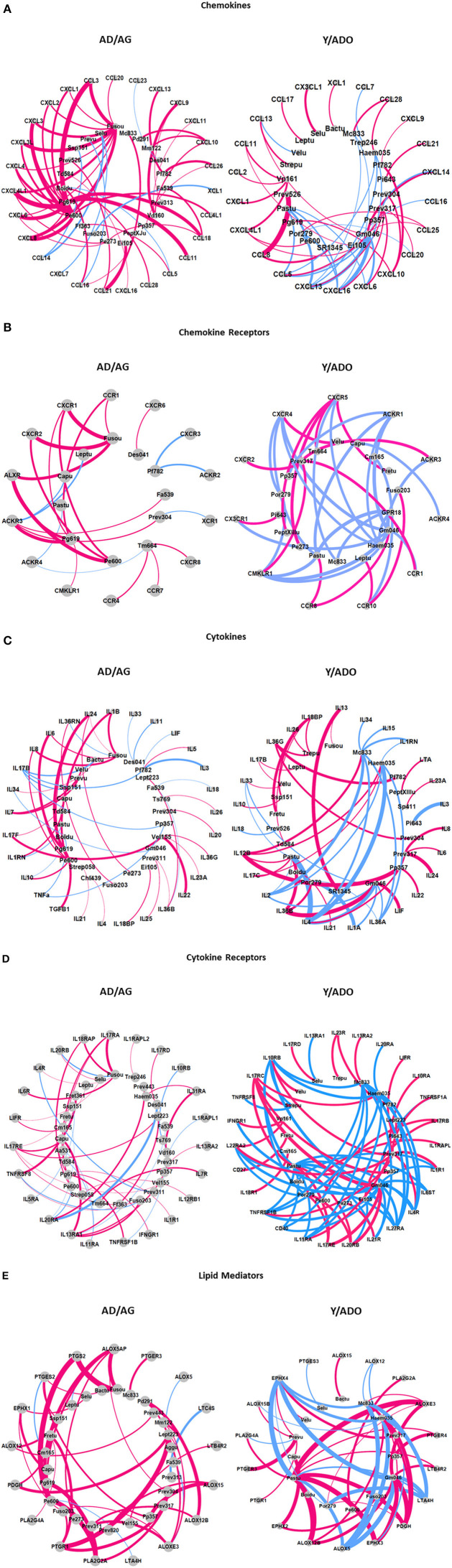
Graphical representation of dominant interactions between microbiome members and chemokine **(A)**, chemokine receptor **(B)**, cytokine **(C)**, cytokine receptor **(D)**, and lipid mediator **(E)** genes in the pooled younger (Y/ADO) and older (AD/AG) group of animals. Magenta lines denote positive correlations and blue denote negative correlations. The thickness of the connections to the nodes signifies the frequency of the interactions between two nodes.

An important concept of the oral microbiome homeostasis or dysbiosis in periodontitis is that the microorganisms demonstrate ecological relationships that reflect cognate interactions, nutritional cooperation or competition, and potential virulence determinants that modify the local microenvironment. Thus, we examined the features of the oral microbiomes in the younger and older age groups focusing on identifying microbes that demonstrate similar patterns of interactions to the array of inflammatory mediators in tissues from juxtaposed sites. Figures 5A–E summarizes these results for the AD/AG and Y/ADO groups for each of the subsets of inflammatory mediator genes. The pattern of these interactions in the AD/AG samples showed strong positive relationships between an array of the bacteria and the chemokines (Figure 5A), represented strongly by *Fusobacterium*_unclassified and *P. gingivalis* HMT619. More limited negative correlations were observed. In contrast in the Y/ADO samples, the majority of the interactions were negative correlations. The interaction patterns with the chemokine receptors also demonstrated a dramatic difference with dominant positive correlations in the AD/AG and negative correlations in the Y/ADO samples (Figure 5B). In the AD/AG samples, fewer bacteria were represented, albeit, *Fusobacterium*_unclassified, *P. gingivalis* HMT619, and *P. enoeca* HMT600 were major representatives. In the Y/ADO samples, the pattern suggested a predilection for selected receptors (e.g., CXCR4, CXCR5, ACKR1, CMKLR1, and CCR10) to be associated with multiple bacteria. In both the AD/AG and Y/ADO samples multiple bacterial OTUs were related to the cytokines in the gingival tissues (Figure 5C). As with the other inflammatory genes types, the majority of the correlations were positive in the AD/AG samples. In the Y/ADO samples, robust negative interactions between an array of the cytokines and *M. catarrhalis* HMT833, *Haemophilus* sp. 035 *G. morbillorum* HMT046, and *Pasturellaceae*_unclassified were seen. Figure 5D showed striking differences between the bacteria and the cytokine receptors between the age groups. A large number of OTUs showed rather limited positive interactions with individual receptors in the AD/AG samples. In contrast, a more limited group of bacteria were broadly correlated with these receptors in the Y/ADO samples. In particular, IL10RB, IL17RC, IL22RA2, TNFRSR1B, IL11RA, IL20RB, IL27RA, IL4R, and IL1R1 showed extensive interactions with *M. catarrhalis* HMT833, *Haemophilus* sp. 035 *G. morbillorum* HMT046, *Pasturellaceae*_unclassified, and *Prevotella* sp. HMT317, similar to the patterns with the cytokine genes. Finally, Figure 5E summarizes the interactions of the OTUs with the lipid mediator genes. As was noted with the other inflammatory genes, positive correlations dominated in the AD/AG samples. *Fusobacterium*_unclassified, *P. gingivalis* HMT619, *P. enoeca* HMT600, and *Leptotrichia* sp. HMT223 demonstrated the most robust interaction across this gene family including ALOX5AP, PTGS2, PTGR1, PLA2G2A, and ALOXE3. A more limited group of bacteria showed interactions in the Y/ADO samples, with a mixture of strong positive and negative correlations with EPHX4, ALOXE3, LTA4H, PDGH, EPHX3, ALOX5, and ALOX12B. As with other inflammatory genes in this age group, the related OTUs highlighted *M. catarrhalis* HMT833, *Haemophilus* sp. 035 *G. morbillorum* HMT046, and *Pasturellaceae*_unclassified.

**Figure 6:**
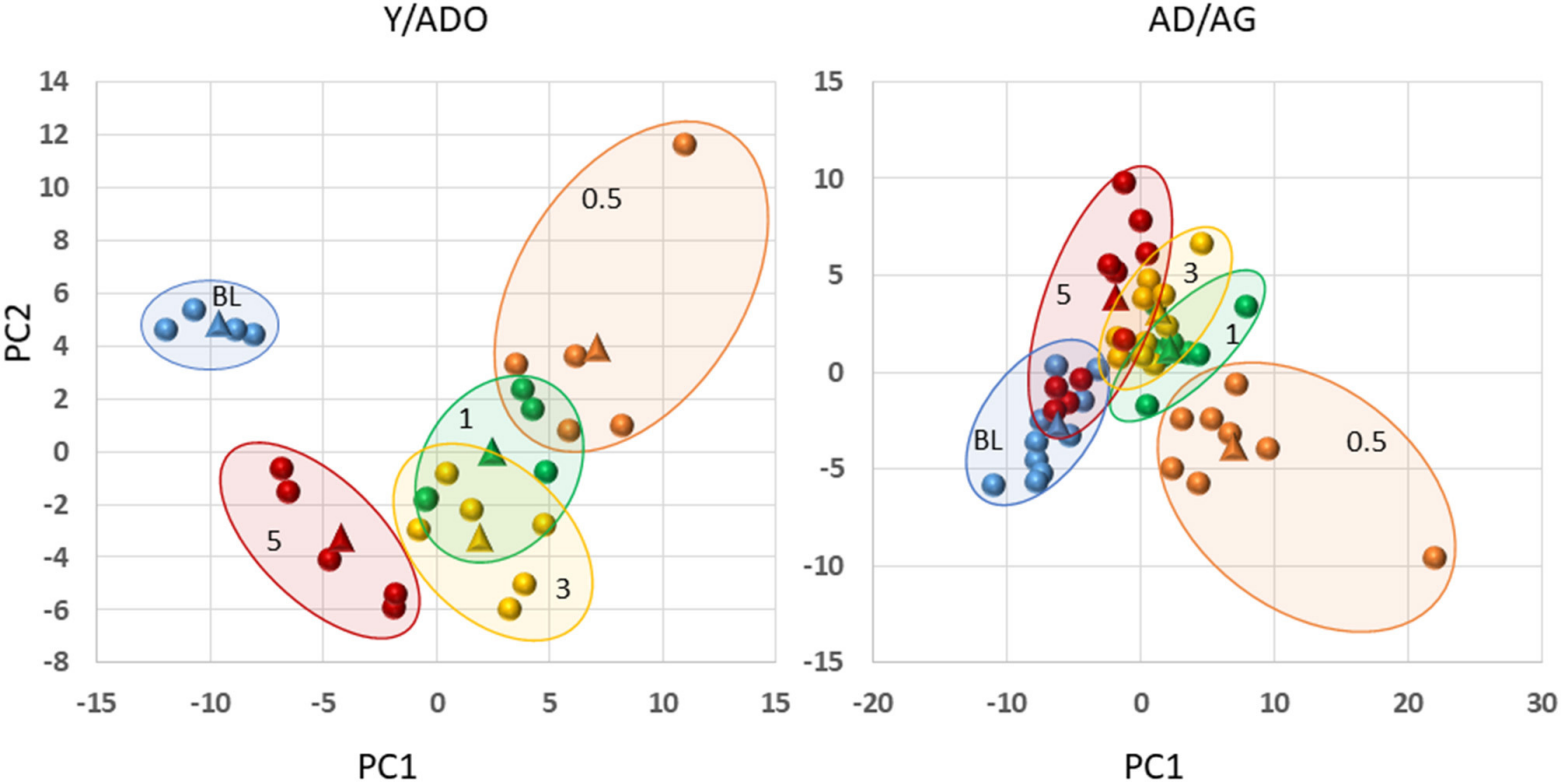
Principal components analysis combining filtered gene expression (*p* < 0.05) and microbiome relative abundance levels in younger (Y/ADO) and older (AD/AG) animals. Each point denotes one specimen at the different time points (BL, health; 0.5, initiation; 1, early progression; 3, late progression; 5, resolution). The triangles signify the mean values for all the samples within the particular time point.

Finally, we examined the capacity of the microbiomes and inflammatory mediator panel to discriminate health, disease initiation/progression, and resolution in the two age groups of animals. Figure 6 displays the results and demonstrates that a clear separation with nearly 50% of the variance in the data was accounted for by the first two principal components. In particular, the baseline (health) and the resolution samples were distinct. Additionally, with these markers, the disease initiation samples (0.5 months) also showed some differential composition; however, the specimens during disease progression (1 and 3 months) were not particularly distinct based upon inflammatory mediators and the microbiome measures. In contrast, in the older group 41% of the variance in the data was accounted by the first two principal components, with the baseline values distinct from all the disease timepoints, and the disease initiation samples (0.5 months) being unique. Minimal differences were seen in the disease progression samples. An additional observation was that while the resolution values were somewhat distinct from the other time points, some of these samples appeared similar to the healthy specimens, and a second subset appeared more closely aligned with the late disease progression values.

## Discussion

Clear evidence is available regarding the initiation and development of the complex microbiome in the oral cavity. Existing data have demonstrated unique features of the microbiome in different niches in the oral cavity [3, 39, 40], periodontitis vs. healthy periodontium in adults demonstrating the effect of smoking and diabetes on the microbiome [2, 5, 41–45], progressing vs. non-progressing periodontal sites [46–48], and distribution of the large array of species across human populations [5, 49]. Chronic periodontal infections provide a very different “face” to the host with regards to controlling these bacteria. Nevertheless, this dynamic host–bacterial environment has evolved to help sustain a protective commensal microbial ecology, while minimizing the ability of opportunistic pathogens to emerge within these established biofilms [50, 51]. As importantly, current concepts of the transition of the ecology from health to disease now emphasize features that propel changes in the balance of microbes, resulting in a “dysbiosis” that can trigger a disease process [52]. It still remains ill-defined how this dysbiosis is created, and whether this is primarily being driven by the emergence of pathogenic bacteria in the subgingival ecology and/or stimulating a dysregulated host innate and inflammatory response that is modulated by genetic and epigenetic predisposition, as well as patient-modifiable factors including smoking, diet, diabetes, stress, etc. [52].

An additional critical component in this environment is clear evidence of the induction of features of both acute and chronic inflammation with the transition in the microbiome and health to disease. A wide array of studies have documented in oral tissues and fluids numerous host innate and inflammatory mediators and cells that differentiate health from disease in humans [9, 53, 54]. However, these data are generally derived from cross-sectional point-in-time studies that demonstrate significant differences. Recognizing the episodic nature of periodontal lesions with exacerbations and remissions suggests a dynamic process of transition from health to disease initiation, followed by disease progression, and eventually lesion resolution. We have previously identified an array of genes that form a profile of responses coincident with these various phases using a non-human primate of ligature-induced periodontitis [55]. Although the patterns of genes identified were derived from a range of biologic pathways, there was a clear representation of innate immune and inflammatory genes. However, somewhat lacking from oral sciences knowledge is the role that the members of the acquired microbiome play in the portfolio of these responses in the oral cavity.

This study focused on a large array of chemokine, chemokine receptor, cytokine, cytokine receptor, and lipid mediator genes in the gingival transcriptome related to periodontitis and modified by age. Existing data stipulate that an altered microbiome drives a dysregulation of the host response resulting in the collateral damage of the periodontal tissues *via* the actions of a wide array of host biomolecules. However, the findings from human studies do not provide a robust kinetic analysis of the detailed temporal changes in the microbiome and host responses that likely occur in days, whereas human studies have been limited to comparisons over multiple-month intervals [56]. Thus, the true dynamic interactions between members of the oral microbiome and the varied host response components that maintain homeostasis or enable a disease process remain somewhat enigmatic. The broader results from this study demonstrate significant interactions between individual members and complexes of oral bacteria with somewhat unique aspects of the inflammatory response system. The non-human primate microbiomes showed approximately 307-394 OTUs that could be detected in at least one sample across the animals [29]. However, we identified that 58 OTUs covered 80–82% of the total OTU reads in the AD/AG samples, and 49 OTUs covered 87–90% of the reads in the Y/ADO samples. Within this pathogenesis model of periodontitis, we observed 15 OTUs with strong positive correlations across multiple subsets of the inflammatory response mediators in the AD/AG animals. This included *Capnocytophaga, Desulfovibrio, Filifactor, Fusobacteria, Leptotrichia, Prevotella, Selenomonas, Pyramidobacter, Treponema*, and *Veillonella*. A major difference was noted in this comparison with the samples from the Y/ADO samples, where 12 OTUs showed robust negative interactions with an array of inflammatory mediators represented by *Bacteroides, Fretibacterium, Gemella, Haemophilus, Moraxella, Pasteurella, Prevotella, Pyramidobacter, Selenomonas*, and *Veillonella*. Therefore, in this model there appears to be some specificity to the microbial taxa most specifically related to the regulation of inflammatory responses. Additional studies will be required to drill down more deeply into the capabilities of individual species, as well as the temporal nature of these interactions.

The overall expression of the chemokines/cytokines and chemokine/cytokine receptors was rather similar in healthy gingival tissues from young to aged animals. This is consistent with an important homeostatic regulation of the array of local gingival inflammatory responses to the septic oral environment throughout the lifespan in a healthy periodontium. However, gene expression profiles for the lipid mediators differed substantially with younger specimens showing decreases in genes related to the synthesis of inflammatory prostaglandins and leukotrienes, as well as effects on prostaglandin inactivation and endogenous resolving lipids. Increased expression levels of ALOXA3, ALOX12B, PTGR2, PTGER3, and EPHX2 are associated with metabolism and cellular receptors for prostaglandins and enzymes contributing to barrier functions of the epithelium. As such, it appears that an array of components of the pathway regulating the inflammatory lipids are expressed differently in young vs. older samples and may relate to the characteristics of periodontal inflammation generally unrelated to tissue destruction in young individuals [57, 58].

The profiles of altered chemokines and chemokine receptors with disease initiation and progression were also generally similar across the age groups. In particular CCL18 (macrophage inflammatory protein 4), CXCL6 (granulocyte chemotactic protein 2), CXCL8 (IL-8), and CXCL13 (B cell-attracting chemokine 1) were elevated and suggest a broad change in the milieu-attracted multiple inflammatory cell types into the lesions. In contrast, CXCL10 (IP-10) and CXCL11 (I-TAC) levels were decreased in young, adolescent, and aged animals with disease progression potentially reflecting altered T cell communication in the developing lesions. A similar outcome was noted with the chemokine receptors, with CCR1 (MIP-1α receptor) and CXCR1 (IL-8 receptor) uniquely increased in all age groups throughout the disease, potentially altering local interactions of resident and inflammatory cells during disease.

Examination of cytokine gene levels showed that IL1B, IL20 (proinflammatory and epithelial function), IL24 (MDA7; member of IL-10 family), and IL33 (member of IL-1 family, signals through IL1RL1; acts on Th2 cells) were increased in all age groups and IL6 only in adults with the disease. Interestingly, IL1B was decreased in the adolescent, adult, and aged animals late in the disease and in lesion resolved tissues. Conversely, IL36B (IL1F8), IL36G (IL1F9), and IL36RN were substantially decreased in the adult and aged animals, and only IL36B was decreased in young and adolescent samples. IL36B and IL36G signal through IL1RL2 receptor and are stimulated in epithelial cells by various inflammatory cytokines as part of the epithelial barrier protection. IL36RN (IL36 receptor antagonist) inhibits the activities of all the members of the IL36 cytokine family and is also considered a part of the epithelial barrier protection. Few cytokine receptor genes were increased in the various age groups. However, IL18RAP, an accessory subunit for the IL-18 proinflammatory receptor, IL22RA2 (inhibits IL22 activity), and IL7R that have a critical role in V(D)J recombination events were increased primarily with disease initiation.

Differences noted in the lipid mediator gene expression in healthy gingiva were also seen in the specimens during disease initiation and progression. ALOX12B, HPGD, PTGS2, and ALOXE3 were all decreased across all age groups and even in resolution samples. As noted these gene products are critical components of the production and regulation of prostaglandins and leukotrienes in inflamed tissues, supporting a contribution to the changes in the local environment following lesion induction.

The abundance of various OTUs in the oral microbiome in health and during disease was also evaluated related to this portfolio of inflammatory genes in the juxtaposed gingival tissues. Specific OTUs of genera and species of oral bacteria were significantly positively or negatively correlated with the inflammatory genes. This was particularly evident in samples from healthy periodontal sites in both the younger and the older animals. Of interest was that the OTUs were mutually exclusive in either positive or negative correlations. Moreover, the individual OTUs showed some specificity regarding the particular family of inflammatory genes dominating in these correlations. Additionally, the dominant OTUs with these correlations were virtually unique between the younger and older specimens. Each of these features provides evidence of a more direct linkage between selected individual members of the oral microbiome and characteristics of the array of inflammatory mediator responses occurring in the gingival tissues. Substantial work has been done in attempting to identify specific components on a small set of oral bacteria that would likely contribute to changes in inflammatory responses in the oral cavity. These include more classic factors such as LPS, fimbriae, outer membrane proteins, exotoxins, proteases, etc. [59–66]. However, generally these studies have focused on microbes historically considered as likely periodontal pathogens. These types of bacteria were identified within the list of genera/species correlated with inflammatory gene expression in this investigation, particularly in disease samples from adult/aged animals and to a more limited extent in the young/adolescent specimens. There also existed a range of bacteria that would be considered members of the non-pathogenic commensal ecology that strongly correlated with the families of inflammatory genes in healthy samples from the young/adolescent animals. In contrast, in healthy adult/aged samples, positively correlated bacteria tended toward the species associated with pathogenic biofilms, whereas the negatively correlated OTUs appeared more consistent with the commensal species. Thus, the results support a very dynamic interaction of the microbiome and gingival tissue responses, as unique feature of this relationship in health and during the disease process. A better understanding of the features of the broader microbiome members could provide some interesting insights into microbial components that may actually trigger a response that enhances homeostasis through selective inflammatory biomolecules and helps regulate the emergence of more pathogenic species.

Multiparameter analyses demonstrated robust interactions between specific members of the oral microbiome and detailed features of the host responses within these five families of inflammatory genes. Of note in these assessments was that the bacteria involved in these interactions were quite distinct in the samples from the younger vs. the older animals. Additionally, the interactions in the adult/aged specimens were generally positive correlations supporting the role of these particular bacteria in contributing to the local inflammatory environment, whereas the interactions in the young/adolescent samples were skewed toward negative correlations. While examination of the overall microbiome demonstrated extensive similarity in the OTUs featured in the different age groups, our previous results showed significant differences in the relative abundance of various bacteria in the younger vs. older microbiomes in both health and disease [24, 29, 67, 68]. These findings extend the age differences by documenting significant distinctions in the host responses to these microbes presenting the “same” stimulatory features but resulting in divergent inflammatory gene expression profiles. The data were also explored to determine the capacity of this set of host genes and the accompanying microbiome to differentiate patterns for individual specimens related to health, phases of the disease process, and with lesion resolution. The results demonstrated clearly different features for health and resolution in the young/adolescent groups. There was less discrimination between the early and late progression samples suggesting that these inflammatory markers were not critical determinants during these phases of disease progression. Similarly, differences in health and resolution samples were seen in the adult/aged samples. However, of note was that the resolution specimens appeared to show a subset that grouped with the healthy baseline samples, and a second subset that retained features more consistent with those of late disease progression. This could infer that certain inflammatory and microbiome features of particular lesion sites that appear clinically resolved, and potentially related to individual animal genetics, retain biologic features of the disease that increase the risk for the future disease at these same sites, as has been shown in humans with periodontitis [69–71]. Additionally, limited separation was observed with early- and late-disease progression samples in the adult/aged samples, supporting that these inflammatory features may not be critical components during this phase of the disease. Finally, the distribution of the specimens obtained at disease initiation showed distinctive features in both age groups of animals. As many of these host response markers have been consistently identified in innate immune and inflammatory responses, these findings support a strong role for their expression in the earliest phases of the periodontitis lesion across the lifespan. These data, in conjunction with our previous studies in the non-human primate disease model, support portfolios of genes and pathways that wax and wane during the disease process closely linked to changes in the bacterial complexes in the microbiome and provide some new insights into the underlying dynamics of the biological reactions in health and disease.

## Data Availability Statement

The datasets presented in this study can be found in online repositories. The names of the repository/repositories and accession number(s) can be found below: https://www.ebi.ac.uk/metagenomics/, E-MTAB-1977 https://www.ncbi.nlm.nih.gov/, GSE180588.

## Ethics Statement

The animal study was reviewed and approved by Institutional Animal Care and Use Committees (IACUC) of the University of Puerto Rico and University of Kentucky.

## Author Contributions

JE and OG were responsible for the design, conduct of the experiment, clinical data and sample collection, interpretation of the data, and preparation of the manuscript. SK was responsible for the preparation of the tissue specimens for analysis and the microbiome samples. RN provided data analytics, interpretation of the data and review of the manuscript. All authors contributed to the article and approved the submitted version.

## Funding

This work was supported by National Institute of Health grant P20GM103538. We express our gratitude to the Caribbean Primate Research Center (CPRC) supported by grant P40RR03640, specifically Drs. Janis Gonzalez Martinez, Luis Orraca, and Armando Burgos, and the Center for Oral Health Research in the College of Dentistry at the University of Kentucky. We also thank the Microarray Core of University Kentucky for their invaluable technical assistance, and Dr. A. Stromberg for initial normalization of the microarray data. We also thank the Genomic Core Laboratory of University Kentucky for their invaluable technical and data management assistance. The authors acknowledge no conflict of interest with the content of this report.

## Conflict of Interest

The authors declare that the research was conducted in the absence of any commercial or financial relationships that could be construed as a potential conflict of interest.

## Publisher's Note

All claims expressed in this article are solely those of the authors and do not necessarily represent those of their affiliated organizations, or those of the publisher, the editors and the reviewers. Any product that may be evaluated in this article, or claim that may be made by its manufacturer, is not guaranteed or endorsed by the publisher.
