# Supplementary figures and images for: Oral Microbiome and Gingival Gene Expression of Inflammatory Biomolecules With Aging and Periodontitis
Source: Front Oral Health. 2021 Sep 17;2:725115. doi: 10.3389/froh.2021.725115 (PMC8757787; doi:10.3389/froh.2021.725115)

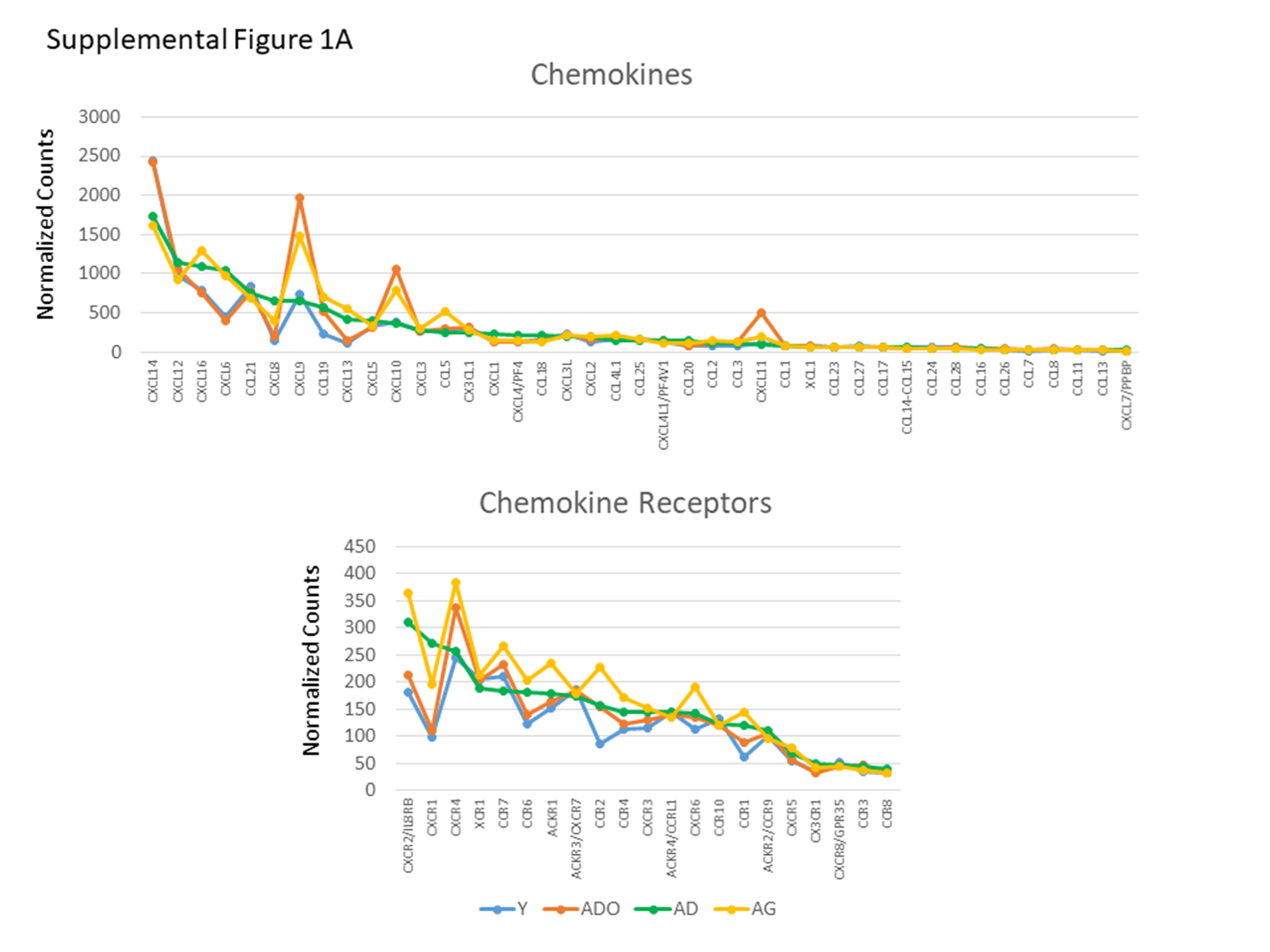

Supplement: Supplementary file 1 [file Image_1.TIF]

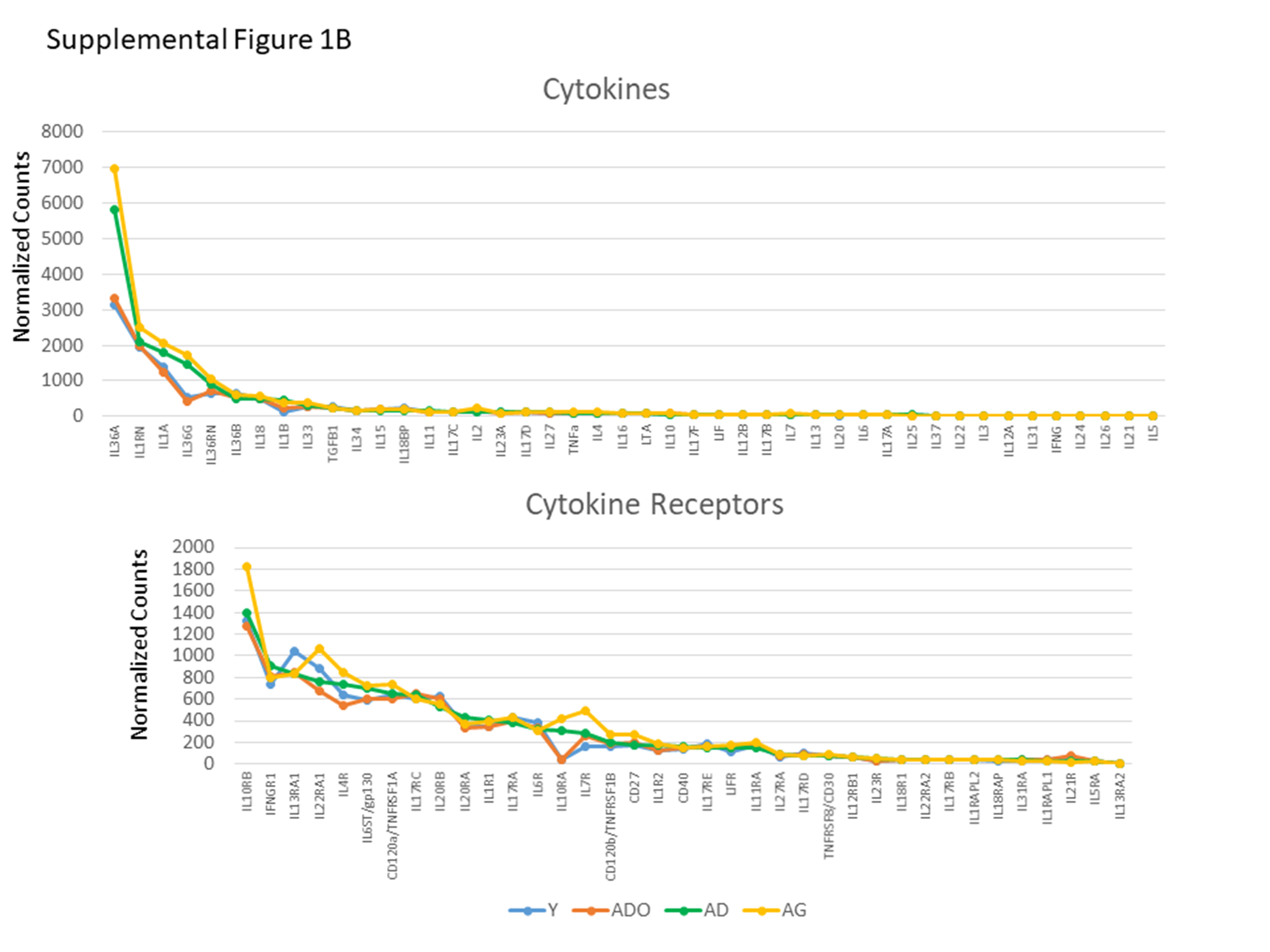

Supplement: Supplementary file 2 [file Image_2.TIF]

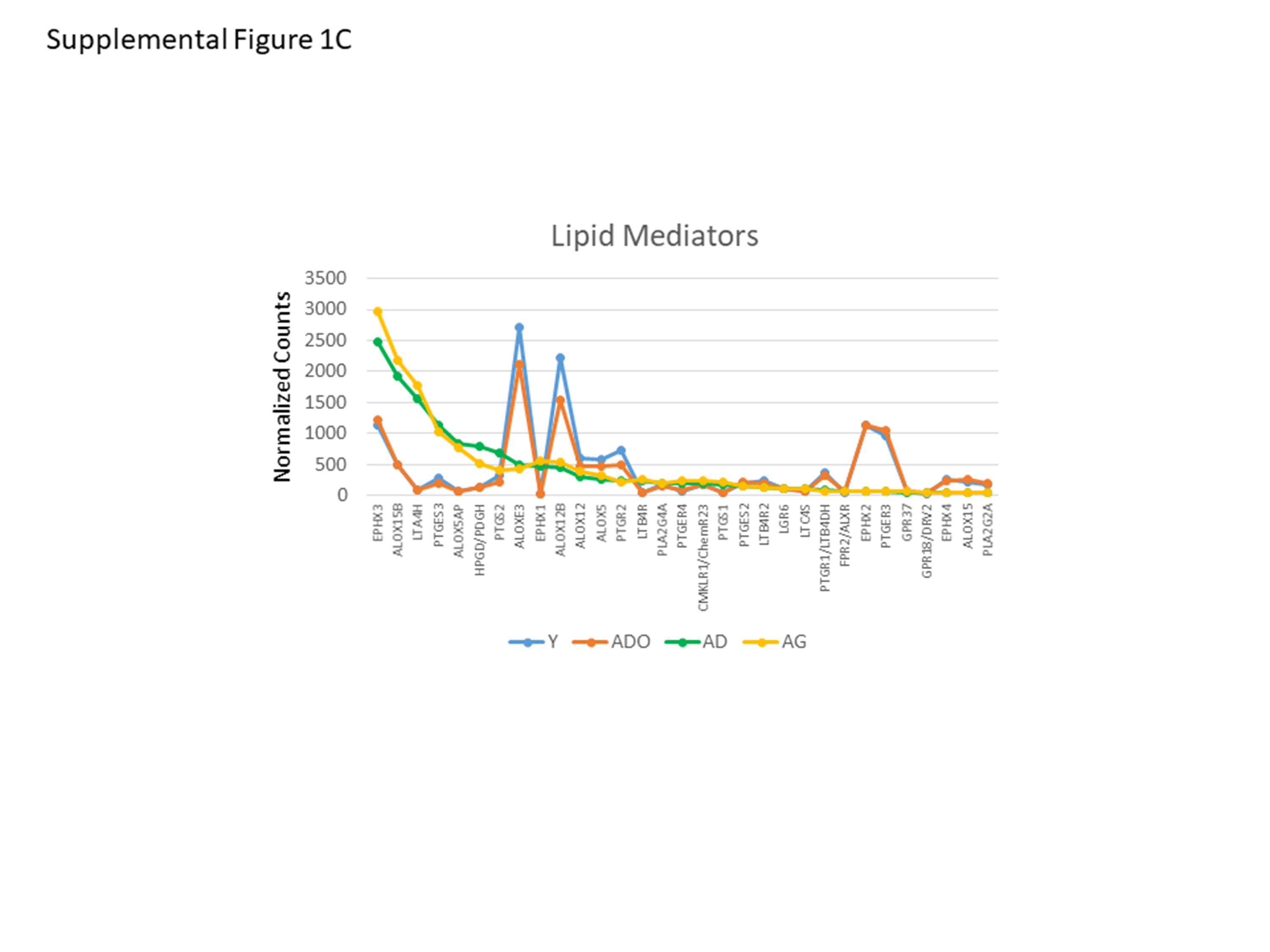

Supplement: Supplementary file 3 [file Image_3.TIF]
