# Supplementary material for: Oral Microbiome and Gingival Gene Expression of Inflammatory Biomolecules With Aging and Periodontitis
Source: Front Oral Health. 2021 Sep 17;2:725115. doi: 10.3389/froh.2021.725115 (PMC8757787; doi:10.3389/froh.2021.725115)
Supplement: Supplementary file 4 [file Data_Sheet_1.docx]

**Supplemental Table 1**: Affymetrix Rhesus GeneChip 1.0 probes and gene IDs.

| **CHEMOKINES** | | **CYTOKINE RECEPTORS** | | 13626348 | IL26 |
| --- | --- | --- | --- | --- | --- |
| 13678144 | CCL1 | 13622769 | CD120a/TNFRSF1A | 13822078 | IL27 |
| 13671787 | CCL11 | 13580850 | CD120b/TNFRSF1B | 13762607 | IL3 |
| 13671796 | CCL13 | 13616036 | CD27 | 13628804 | IL31 |
| 13678299 | CCL14-CCL15 | 13610718 | CD40 | 13667895 | IL33 |
| 13819822 | CCL16 | 13742430 | IFNGR1 | 13724576 | IL34 |
| 13719231 | CCL17 | 13652323 | IL10RA | 13641252 | IL36A |
| 13671907 | CCL18 | 13732599 | IL10RB | 13645890 | IL36B |
| 13662586 | CCL19 | 13667351 | IL11RA | 13641244 | IL36G |
| 13671776 | CCL2 | 13699169 | IL12RB1 | 13641256 | IL36RN |
| 13632412 | CCL20 | 13807672 | IL13RA1 | 13641235 | IL37 |
| 13662581 | CCL21 | 13811907 | IL13RA2 | 13701567 | IL4 |
| 13678304 | CCL23 | 13607498 | IL17RA | 13767994 | IL5 |
| 13727629 | CCL24 | 13711452 | IL17RB | 13735121 | IL6 |
| 13697753 | CCL25 | 13710734 | IL17RC | 13792282 | IL7 |
| 13727633 | CCL26 | 13704695 | IL17RD | 13631846 | IL8 |
| 13662593 | CCL27 | 13710739 | IL17RE | 13614112 | LIF |
| 13766180 | CCL28 | 13640772 | IL18R1 | 13739117 | LTA |
| 13678315 | CCL3 | 13640784 | IL18RAP | 13718598 | TGFB1 |
| 13671917 | CCL4L1 | 13640727 | IL1R1 | 13739126 | TNFA |
| 13678276 | CCL5 | 13640715 | IL1R2 | **LIPID MEDIATORS** | |
| 13671781 | CCL7 | 13805026 | IL1RAPL1 | 13676699 | ALOX12B |
| 13671791 | CCL8 | 13807194 | IL1RAPL2 | 13670444 | ALOX15B |
| 13813522 | CX3CL1 | 13742445 | IL20RA | 13795672 | ALOX5 |
| 13756397 | CXCL1 | 13714816 | IL20RB | 13682757 | ALOX5AP |
| 13751757 | CXCL10 | 13718087 | IL21R | 13600881 | EPHX1 |
| 13751751 | CXCL11 | 13594011 | IL22RA1 | 13787075 | EPHX2 |
| 13800759 | CXCL12 | 13742439 | IL22RA2 | 13698849 | EPHX3 |
| 13756250 | CXCL13 | 13584450 | IL23R | 13585135 | EPHX4 |
| 13768204 | CXCL14 | 13692132 | IL27RA | 13758930 | HPGD/PDGH |
| 13676206 | CXCL16 | 13760382 | IL31RA | 13627401 | LTA4H |
| 13813762 | CXCL2 | 13718075 | IL4R | 13774695 | LTB4R/BLT1 |
| 13751868 | CXCL3 | 13704129 | IL5RA | 13774692 | LTB4R2 |
| 13814192 | CXCL3L | 13587273 | IL6R | 13764897 | LTC4S |
| 13751880 | CXCL4/PF4 | 13766405 | IL6ST/gp130 | 13593639 | PLA2G2A |
| 13756399 | CXCL4L1/PF4V1 | 13759870 | IL7R | 13602451 | PLA2G4A |
| 13815546 | CXCL5 | 13765915 | LIFR | 13596747 | PTGER3 |
| 13756404 | CXCL6 | 13580831 | TNFRSF8/CD30 | 13760040 | PTGER4 |
| 13816774 | CXCL7/PPBP | **CYTOKINES** | | 13664182 | PTGES2 |
| 13756408 | CXCL8 | 13626341 | IFNG | 13661644 | PTGR1/LTB4DH |
| 13751763 | CXCL9 | 13589121 | IL10 | 13776496 | PTGR2 |
| 13603338 | XCL1 | 13822874 | IL11 | 13666092 | PTGS1 |
| **CHEMOKINE RECEPTORS** | | 13714116 | IL12A | 13589962 | PTGS2 |
| 13587845 | ACKR1 | 13769388 | IL12B | 13676714 | ALOXE3 |
| 13712481 | ACKR2/CCR9 | 13762677 | IL13 | 13676169 | ALOX15 |
| 13632876 | ACKR3/CXCR7 | 13799623 | IL15 | 13669935 | ALOX12 |
| 13715001 | ACKR4/CCRL1 | 13773225 | IL16 | 13695837 | FPR2/ALXR |
| 13827254 | CCR1 | 13740835 | IL17A | 13818142 | ERV1/ChemR23/  CMKLR1 |
| 13679947 | CCR10 | 13768920 | IL17B | 13686217 | GPR18/DRV2 |
| 13712469 | CCR2 | 13720894 | IL17C | 13736564 | GPR37 |
| 13712472 | CCR3 | 13820126 | IL17D | 13821766 | LGR6 |
| 13708701 | CCR4 | 13746750 | IL17F |  |  |
| 13827264 | CCR6 | 13658897 | IL18 |  |  |
| 13679479 | CCR7 | 13650624 | IL18BP |  |  |
| 13712956 | CCR8 | 13645870 | IL1A |  |  |
| 13706377 | CX3CR1 | 13645879 | IL1B |  |  |
| 13827278 | CXCR1 | 13641271 | IL1RN |  |  |
| 13631846 | CXCR2/IL8RB | 13614577 | IL2 |  |  |
| 13817340 | CXCR3 | 13601663 | IL20 |  |  |
| 13641372 | CXCR4 | 13757952 | IL21 |  |  |
| 13652473 | CXCR5 | 13626353 | IL22 |  |  |
| 13817268 | CXCR6 | 13618605 | IL23A |  |  |
| 13633079 | CXCR8/GPR35 | 13601652 | IL24 |  |  |
| 13706159 | XCR1 | 13774424 | IL25 |  |  |

**Supplemental Figure 1:** Normalized expression values for chemokine and chemokine receptor (**A**), cytokine and cytokine receptor (**B**), and lipid mediator (**C**) genes in healthy gingival tissues across age groups. Each point denotes the mean value from nine animals/group. Gene order is organized from highest to lowest based upon the expression level in the adult samples.
